# X-ray irradiation promotes apoptosis of hippocampal neurons through up-regulation of Cdk5 and p25

**DOI:** 10.1186/1475-2867-13-47

**Published:** 2013-05-20

**Authors:** Ai-Min Sun, Chuan-Gang Li, Yong-Qing Han, Que-Ling Liu, Qiong Xia, Ya-Wei Yuan

**Affiliations:** 1 Department of Radiation Oncology, Nanfang Hospital, Southern Medical University, Guangzhou, Guangdong, 510515, China; 2 Department of Ultrasound Diagnosis, Nanfang Hospital, Southern Medical University, Guangzhou, Guangdong, China; 3 Medical Imaging, Nanfang Hospital, Southern Medical University, Guangzhou, Guangdong, China; 4 Current Affiliation: Department of Oncology, Shangrao People's Hospital, Shangrao, Jiangxi, China; 5 Current Affiliation: Department of Oncology, Second Hospital, Nanchang University Medical College, Nanchang, Jiangxi, China

**Keywords:** Cdk5, p35, p25, Apoptosis, Irradiation, Hippocampus

## Abstract

**Background:**

Cranial radiation therapy has been used for the treatment of primary and metastatic brain tumors. A prominent feature of brain injury induced by the radiation therapy is hippocampal dysfunction, characterized by a decline in memory. Cdk5 plays an important role in memory formation. Abnormal Cdk5 activity is associated with neuronal apoptosis induced by neurotoxic stimuli. However, the roles of Cdk5 in hippocampal apoptosis in response to X-ray irradiation have not been explored.

**Methods:**

The expression of Cdk5 activators, p35 and p25, in hippocampal neurons was tested in both in vivo animal and in vitro couture after X-ray irradiation.

**Results:**

After X-ray irradiation at 20 Gy and 30 Gy in rats, the number of hippocampal neuronal pyknosis was increased, but the number of hippocampal neuron was decreased, in the hippocampal CA1 region of rats. In these animals undergone with X-ray irradiation, the expression of p35 was significantly down-regulated, but it was up-regulated in p25. These opposite expressions were also shown in the primary cultured hippocampal neurons with 30 Gy irradiation. The apoptosis induced by X-ray irradiation were significantly prevented by the pretreatment of Cdk5 inhibitor, roscovitine, in both in vivo and in vitro settings.

**Conclusions:**

X-ray irradiation resulted in a hippocampal neuronal apoptosis through up-regulation of p25, the Cdk5 activator. Hyperactivity of Cdk5 was involved in the pathogenesis of X-ray irradiation-induced hippocampal neuronal apoptosis. Blockade of Cdk5 signal pathway effectively protected neurons from the irradiation-induced brain injury.

## Background

Cranial radiation therapy is important in the treatment of primary and metastatic brain tumors. Despite its ability to prolong the survival of patients, the use of radiation therapy is limited by toxicity to the normal brain function. Hippocampal dysfunction is a prominent feature of brain injury resulted from radiation therapy, characterized by a progressive decline in the learning, memory, and spatial information processing abilities [1-3]. However, the mechanisms behind the hippocampus-dependent cognitive dysfunction caused by radiation therapy have not been established.

The most commonly reported cellular mechanisms underlying radiation-mediated injury are via breakup of DNA and consequent disruption of many signal pathways involved in cell cycle, apoptosis and stress response [4]. Hippocampus is particularly vulnerable to ionizing radiation. Radiation-induced learning and memory deficits have been reported, and are associated with an increase in hippocampal apoptosis [5,6] and a decrease in neurogenesis [6-8]. It is likely that hippocampal vulnerability may result from cellular components involved in both hippocampal apoptosis and neurogenesis. However, the cellular mechanisms underlying the vulnerability of hippocampus remain to be elucidated.

Cdk5 is highly enriched in the hippocampus, and plays a critical role in memory formation, hippocampal apoptosis and adult hippocampal neurogenesis [9,10]. Therefore, it may be attractive target for radiation-induced hippocampal injury. Cdk5, a proline-directed serine/threonine kinase, plays an important role in the physiological functions such as synaptic plasticity, memory formation [9,11,12], and some diseases including Alzheimer’s disease, Parkinson’s disease, and hippocampal sclerosis [9,13,14]. The activity of Cdk5 depends on its activator p35, which can be cleaved to p25 by calpain. p25, the truncated form of p35, binds and hyper-activates Cdk5, and the hyper-activation of Cdk5 by p25 plays a prominent role in neuro-degeneration [15,16]. In addition, the expression of Cdk5 is up-regulated during neuronal death in response to different toxic stimuli, such as oxidative stress, excitotoxic stimulation, and β-amyloid exposure [15,17-19]. However, the roles of Cdk5 in hippocampal apoptosis and its inhibitor, roscovitine, in protective effects on the memory loss in response to X-ray irradiation have not been explored. In the present study, therefore, we explored the X-ray-induced cell apoptosis and protection of roscovitine from the apoptosis in both in vitro and in vivo hippocampal neurons.

## Results

### Roscovitine protected hippocampal cells from apoptosis caused by X-ray irradiation

**Figure 1:**
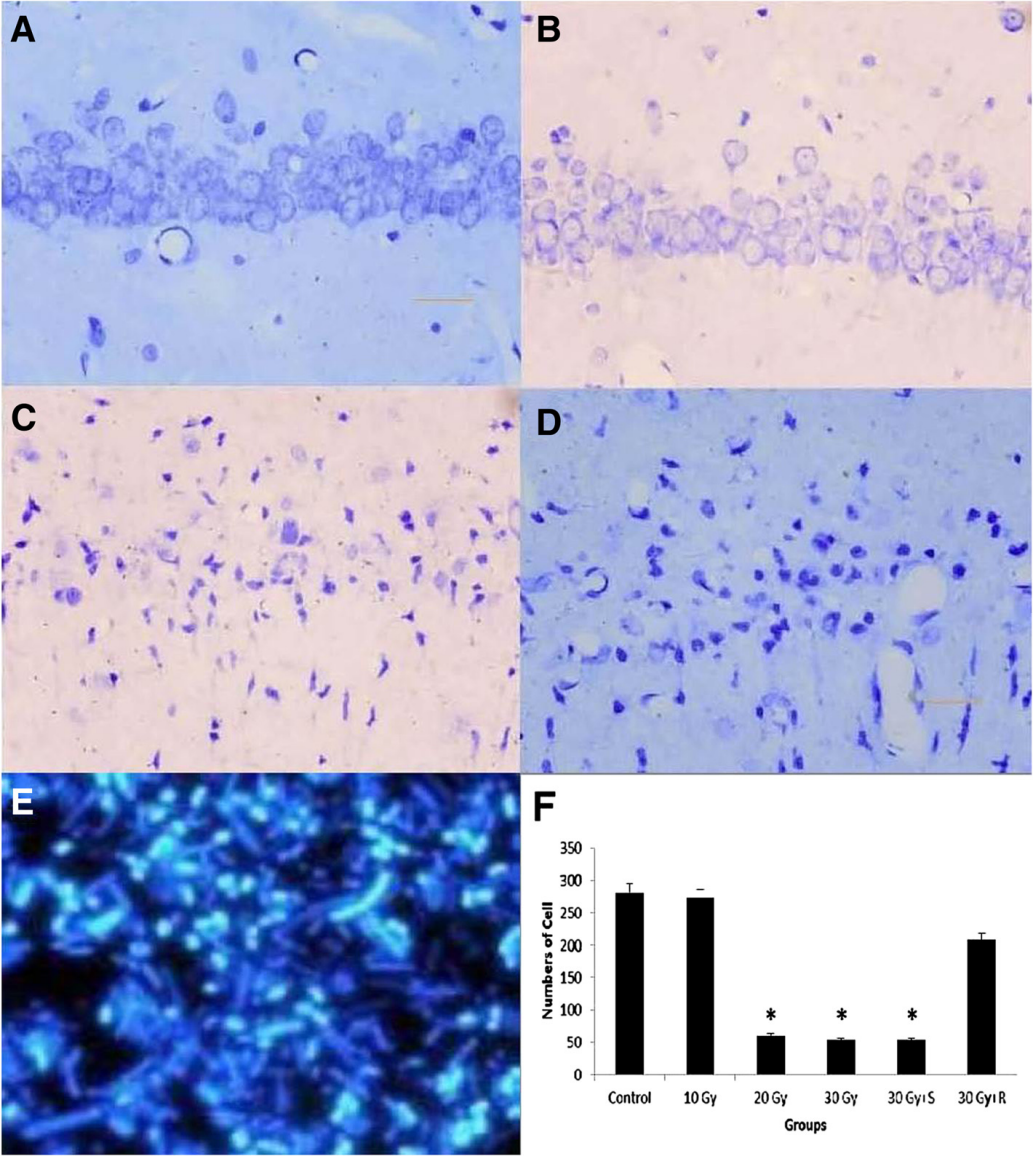
**A**-**F Hippocampal neuronal apoptosis in CA1 region in rats after irradiation.** DAPI fluorescent images showing hippocampal neuronal apoptosis in rats with sham-irradiated control (**A**), irradiation of 10 Gy (**B**), 20 Gy (**C**), 30 Gy (**D**), and 30 Gy+R (**E**). The cells were counted in a 1 mm length of the middle portion of hipocampal CA1 region under bright-filed microscopy. n=6 for each group. *p<0.05 in 20 Gy and 30 Gy vs. control, *p<0.05 in 30 Gy+R vs. 30 Gy+S. R=Roscovitine, S=Saline.

In rats with X-ray irradiation, the number of nuclear pyknosis was increased, while the number of hippocampal cells was decreased, in the group with 20 and 30 Gy irradiation, but not with 10 Gy, compared with that in sham-irradiated control (Figure 1A-D). In rats administrated with roscovitine before X-ray irradiation, the number of hippocampal cells was significantly increased (Figure 1E), compared with the control animals received saline. The control animals and 30 Gy animals were not significantly different in the number of hippocampal cells (Figure 1F). These in vivo and in vitro results suggested that X-ray irradiation caused apoptosis of the hippocampal cells, and that the administration of roscovitine prevented the apoptosis caused by X-ray irradiation (Figure 1A-F).

### p35 and p25 were linked with activation of Cdk5 after X-ray irradiation

**Figure 2:**
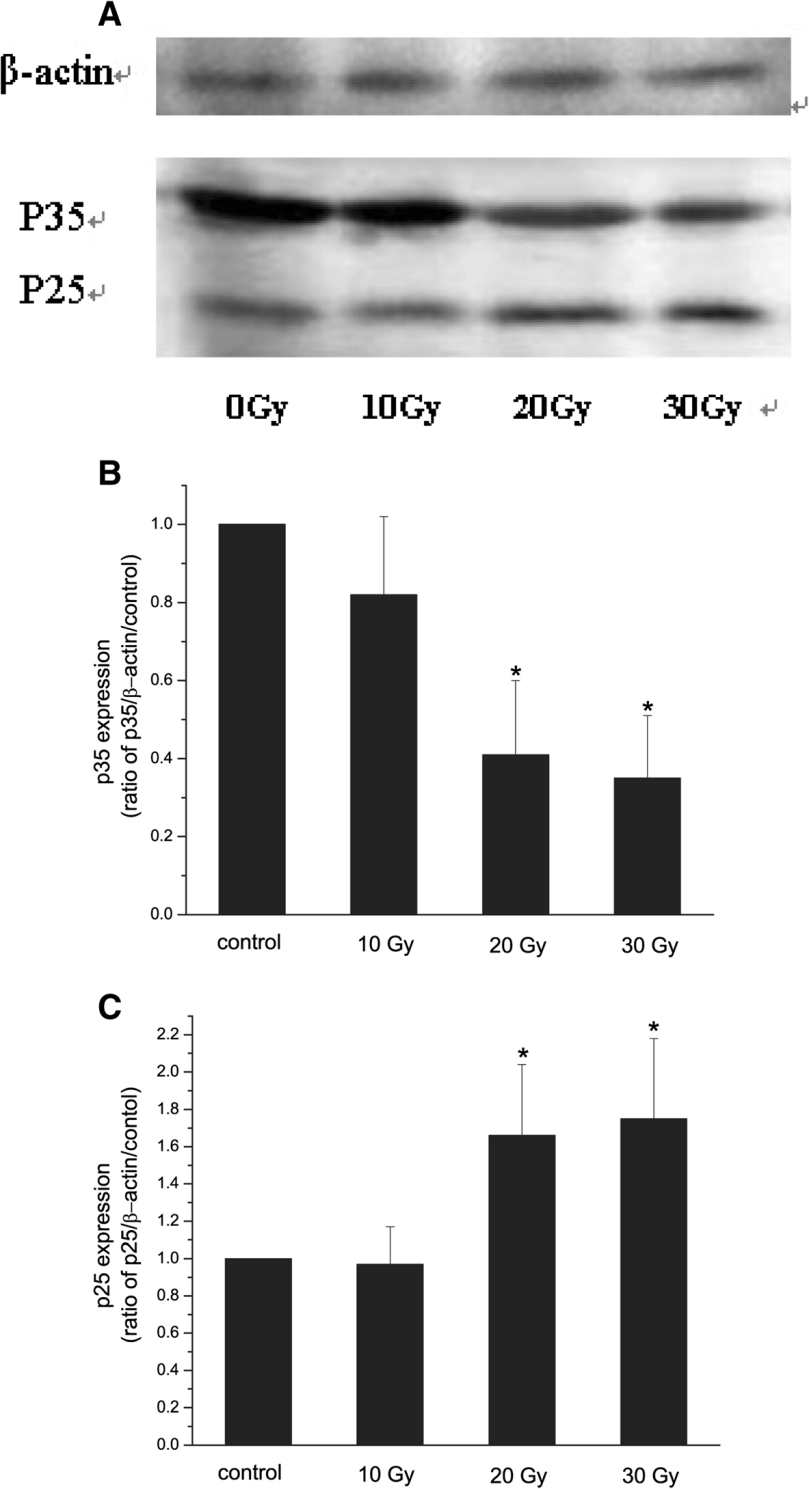
**A**-**C Western blot analysis of p35 and p25 expression in rats after irradiation**. **Representative Western blot showing the expression of p35 and p25 in rat hippocampi.** Lane 1, sham-irradiation; lane 2, 10 Gy irradiation; lane 3, 20 Gy irradiation; lane 4, 30 Gy irradiation (**A**). Quantitative Western blot analysis of p35 (**B**) and p25 (**C**) expression in rats with sham-irradiation. β-actin was a loading control. n=3. *p<0.05 vs. control.

To explore the possible mechanism of hippocampal neuronal apoptosis induced by X-ray, the levels of Cdk5 activators, in vivo p35 and p25, were tested. It was shown that p35 was significantly down-regulated in the group irradiated with 20 and 30 Gy, but not 10 Gy (Figure 2A and B). A significant up-regulation of p25 was found in 20 and 30 Gy, but not 10 Gy (Figure 2A and C). These results suggested that X-ray irradiation up-regulated p25 through an increase in cleavage of p35, resulting in hyperactivation of Cdk5.

**Figure 3:**
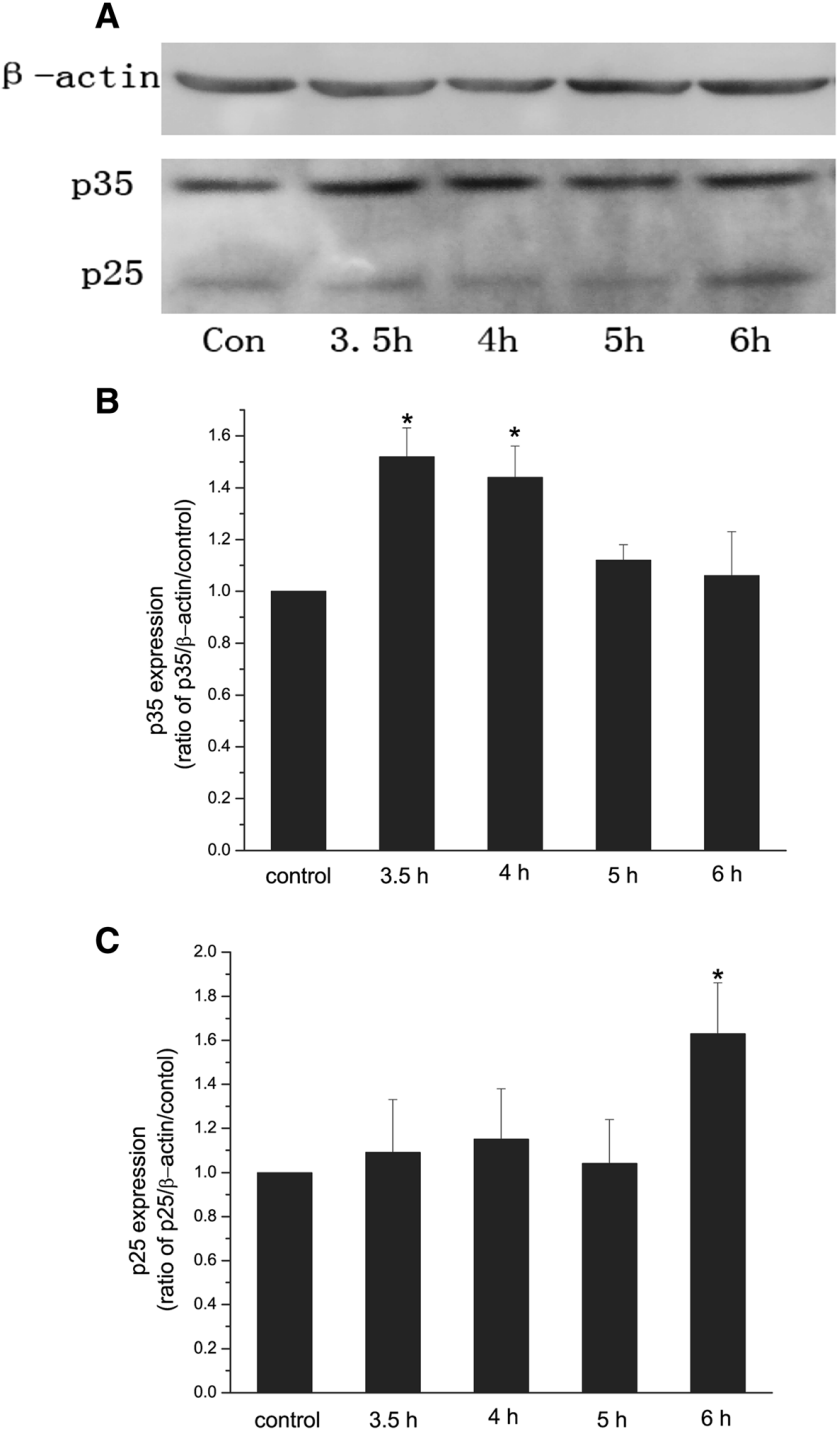
**A**-**C Western blot analysis of p35 and p25 expression in cultured hippocampal neurons at different time after irradiation.** Representative Western blot showing the expression of p35 and p25 in cultured hippocampal neurons exposed to a single dose of 30 Gy irradiation, and tested at 3.5 h, 4 h, 5 h, and 6 h after irradiation (**A**). Expression of p35 and p25 was tested at3.5 h after sham-irradiation as control. Quantitative Western blot analysis of p35 (**B**) and p25 (**C**) expression in cultured hippocampal neurons with sham-irradiation, and with 30 Gy irradiation at 3.5, 4 h, 5 h, and 6 h after irradiation. β-actin was a loading control. n=4. *p<0.05 vs. control.

To further confirm the roles of p25 and p35 in X-ray irradiation-induced apoptosis of hippocampal neurons, their expressions of in vitro hippocampal neurons were tested. Cell cultures were exposed to a single dose of 30 Gy irradiation, and the expressions of p35 and p25 were tested 3.5, 4, 5 and 6 hours after X-ray irradiation. It was shown that the expression of p35 was significantly up-regulated 3.5 and 4 hours after irradiation (Figure 3A and B). The expression of p25 was significantly increased 6 hours after irradiation (Figure 3A and C). The expression of p25 was elevated as the expression of p35 decreased, suggesting that up-regulation of p25 was mediated through the cleavage of p35.

### Cdk5 was involved in hippocampal neuronal apoptosis induced by X-ray irradiation

**Figure 4:**
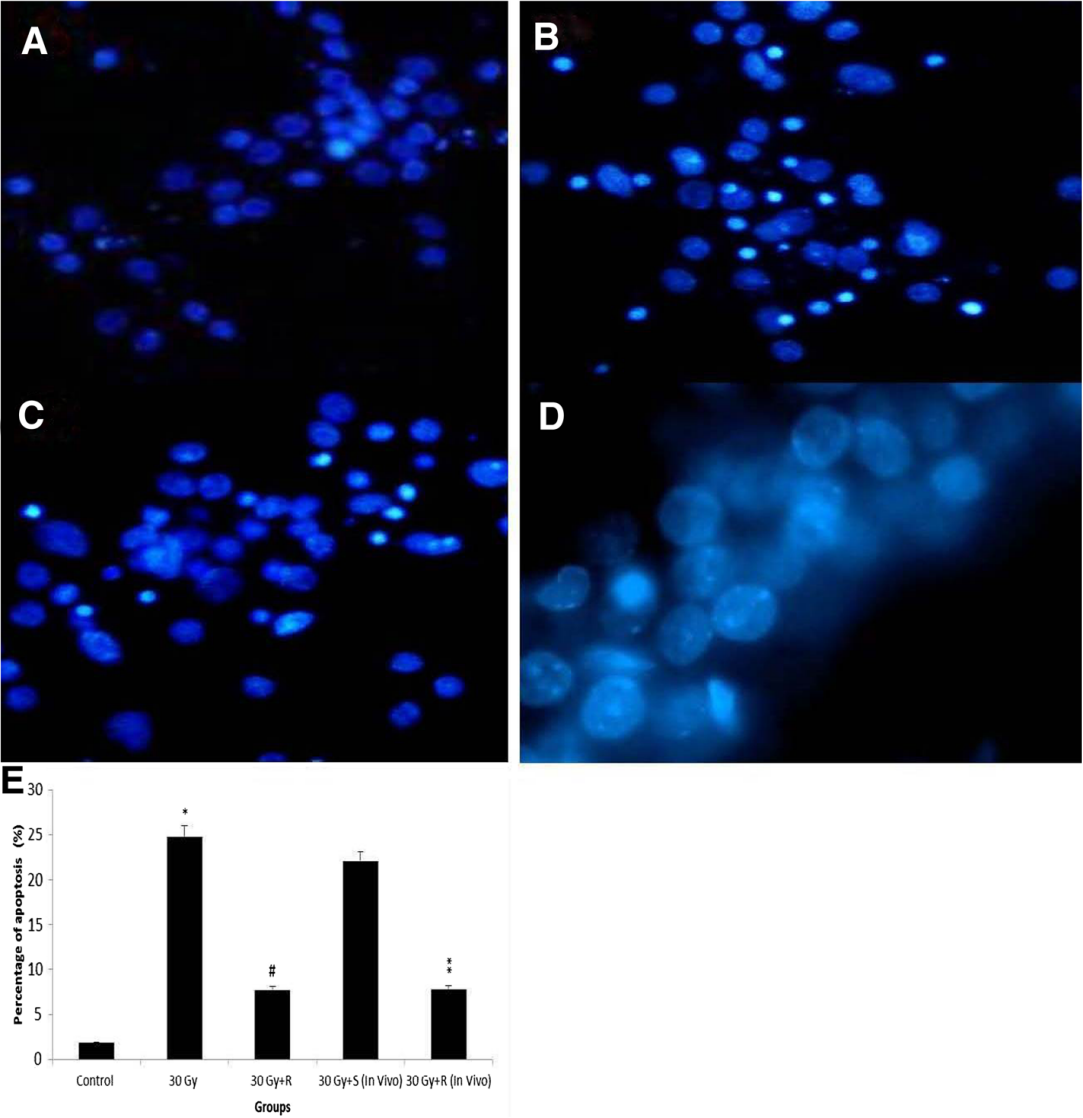
**A-E Hippocampal neuronal apoptosis in both in vitro and in vivo hippocampal neurons after irradiation.** DAPI fluorescent images showing pyknosis of in vitro hippocampal neurons exposed to sham-irradiation (**A**), 30 Gy (**B**), 30 Gy with roscovitine (in vitro) (**C**), and 30 Gy with roscovitine (in vivo) (**D**). Percentage of in vitro and in vivo pyknosis with and without administration of roscovitine (**E**). *p<0.05 vs. control, #p<0.05 vs. 30 Gy, **p<0.05 vs. 30 Gy+S. S=Saline, R=Roscovitine.

To study the role of Cdk5 in apoptosis of hippocampal neurons, both the primary cultured hippocampal neurons and animals were pretreated with Cdk5 inhibitor, roscovitine, and the apoptosis were examined after 30 Gy of irradiation. The nuclear pyknosis of the in vitro hippocampal neurons was increased in the number after the irradiation of 30 Gy, and the decrease in number was reversed by pretreatment of roscovitine (Figure 4A, B, and C). Moreover, the number of in vivo nuclear pyknosis was also increased in the control animals receiving saline, while the number was greatly decreased with administration of roscovitine (Figure 4D). The percentage of apoptotic neurons induced by X-ray irradiation (in vitro 24.8±3.97%, and in vivo 22.1±3.7%) were significantly decreased by administration of roscovitine (in vitro 7.74±2.27%, and in vivo 7.86±1.91%, p<0.05, Figure 4E). These results suggested that hyperactivity of Cdk5 was linked with hippocampal neuronal apoptosis induced by X-ray irradiation, and the administration of roscovitine, in part, protected hippocampal neurons from the irradiation-induced apoptosis.

## Discussion

X-ray irradiation results in neuronal apoptosis via DNA damage, which disrupts signal pathways involved in apoptosis [4]. Many stress insults including oxidative stress [20], excitotoxic stimulation [21,22], and β-amyloid exposure [23] have been reported to induce neuronal apoptosis. Neuronal apoptosis also occur in the neurodegenerative diseases such as Alzheimer’s disease and Parkinson’s disease [24,25]. It has been reported that hyperactivity of Cdk5 is associated with the neuro-degeneration in Alzheimer’s disease and Parkinson’s disease [13,14,26], and plays an important role in neuronal apoptosis resulted from those stress insults [20-23]. However, the role of Cdk5 in X-ray induced-apoptosis remains largely unknown. In this study, therefore, we investigated the expression of Cdk5/p25 in X-ray induced apoptosis in hippocampal neurons. Results from the present study showed that the expression of Cdk5/p25 was up-regulated in hippocampal neurons after X-ray irradiation, and inhibition of Cdk5 activity prevented hippocampal neuronal apoptosis induced by X-ray irradiation.

Cdk5 plays an important role in neuronal migration, axon guidance, cytoskeletal protein phosphorylation, and synaptic transmission [11,12]; activity of Cdk5 is tightly regulated by its neuron-specific activator p35/p39. The breakdown of p35 by calpain into p25, a stable form of p35, increases Cdk5 kinase activity. However, abnormal activation of Cdk5 is toxic to neurons, leading to apoptosis under both physiological and pathological conditions [15,16]. Hyperactivity of Cdk5 has been reported in neurodegenerative disorders including Alzheimer’s disease, Parkinson’s disease and amyotrophic lateral sclerosis [13,14,26]. In agreement with Cdk5 hyperactivity leading to neuronal apoptosis, the expression of p25 was elevated by X-ray irradiation, and the pretreatment of Cdk5 inhibitor, roscovitine, significantly prevented the neuronal apoptosis resulted from X-ray irradiation, suggesting that X-ray irradiation resulted in neuronal apoptosis through hyperactivity of Cdk5.

Several studies have shown that inhibition of Cdk5 can protect neurons from apoptosis. Cdk5 inhibitory peptide, which effectively inhibits Cdk5 kinase activity (29), reduces neuronal apoptosis induced by β-amyloid [27]. Cdk5 inhibition by Cdk5 inhibitor butyrolactone and Cdk5 antisense nucleotides also protects neurons from β-amyloid- mediated cell death [15,28]. In consistent with the idea that inhibition of Cdk5 produces neuroprotective effects, our results showed that pretreatment of roscovitine prevented X-ray irradiation-induced apoptosis in both in vivo and in vitro settings.

It is well known that X-ray irradiation causes cell death via breakup of the DNA [4]. Activation of Cdk5 plays an important role in neuronal death induced by DNA damage [29]. However, it is unclear how Cdk5 is activated after DNA damage. Our study showed that X-ray irradiation resulted in an up-regulated expression of p25 associated with a decrease in p35 expression, suggesting that X-ray irradiation activated Cdk5 through calpain-mediated cleavage of p35 to a more stable p25. Hyperactivity of Cdk5 has been reported to result in neuronal apoptosis [12]. However, the signal pathways leading to the activation of p25 at X-ray irradiation-induced DNA damage need to be elucidated in the future studies.

## Conclusions

X-ray irradiation enhanced the levels of p25 in hippocampal neurons in both in vivo rats and in vitro cultured hippocampal neurons, which suggested that hyperactivity of Cdk5 was involved in the pathogenesis of radiation-induced hippocampal neuron injury. Inhibition of Cdk5 activity by the Cdk5 inhibitor, roscovitine, significantly protected hippocampal neuronal cells from death, which suggested that the blockade of Cdk5 signal pathways was an effective strategy to protect neurons from X-ray-induced brain injury during cranial radiation therapy.

## Methods

The experimental protocols were approved by the Committee for Animal Experiment of the Southern Medical University.

### Animals

Male Sprague Dawley rats (weighing 200±20 g) were used for X-ray irradiation. One-day Sprague Dawley rats were used for hippocampal culture. All rats were obtained from animal center at Southern Medical University. The rats were fed a standard animal diet.

### Intravenous administration of roscovitine

To determine protective effects of roscovitine on hippocampal CA1 neurons in rats, twelve rats were randomly assigned to two groups (n=6 in each group) receiving roscovitine and saline, respectively. Roscovitine was intravenously administrated at dose of 30 mg/kg in a volume of 1mL through a tail vein 30 min before irradiation, and the saline group was intravenously injected in the same volume of saline. Roscovitine was dissolved in dimethyl sulfoxide (Me_2_SO) by following the previously reported method [30].

### X-ray irradiation

Rats were intraperitoneally administrated with 10% chloral hydrate (3.5 ml/100 g body weight), and affixed in a Plexiglass plate in the prone position. Whole body radiation was delivered by linear accelerator (Varian Medical Systems, Inc. Palo Alto, CA, USA) using 12 MeV electron beam at a dose of 400 cGy/min with a source-skin distance of 100 cm. For the sham-irradiated control groups, a lead shield was used to protect rats. The animals, depending on the radiation used, were assigned to three groups: 10, 20, and 30 Gy radiation (n=6 in each group). The animals receiving roscovitine and saline underwent 30 Gy radiation only.

For the primary cultured hippocampal neurons, X-ray irradiation was performed by 6 MeV electron beam at a dose rate of 400 cGy/min. The in vitro cells were undergone with a single dose of 30 Gy, and the control cells acted as a sham-irradiated under the same conditions.

### Hippocampal neuron culture

Hippocampal neurons were prepared from hippocampi of newly born rats as described by Katsube et al. [31]. Briefly, hippocampi were cut into small pieces, and incubated in 0.25% trypsin for 5 min. The hippocampi were then triturated by aspirating about 10 times using a pasterur pipette. The cells were plated in Neurobasal medium on poly-L-lysine coated culture dishes for 72 hours. Cytosine-b-D-arabinofuranoside (5 μM) was added to the culture dishes to inhibit non-neuronal cell proliferation. After 12-day culture, the neurons were identified using phosphorylated neurofilament antibodies. Cells with neuronal purity of more than 90% were used for the experiments.

### Western blot

For Western blot analysis, hippocampal tissue was dissected from the brain. The hippocampal tissue or cultured hippocampal neurons were homogenized on ice in lysis buffer. Proteins were separated by electrophoresis in 10% SDS–PAGE, and transferred onto polyvinylidene fluoride membranes by electroblotting. Membranes were rehydrated with methanol and incubated in buffer (5% powdered milk in PBS) for 30 min. Membranes were incubated with primary antibodies against p35/p25 (C-19, dilution 1:1000, Cell Signaling, USA) or primary antibodies against β-actin (dilution) at 4°C overnight. Blots were developed using horseradish peroxidase-linked secondary antibodies (dilution 1:5000) and a chemiluminescent detection system as recommended by the manufacturer.

### Quantification of apoptosis

Rats were intraperitoneally administrated by 10% chloral hydrate (3.5ml/100 g), and were perfused with 4% formaldehyde via the left ventricular. The brains of the animals were dissected, and post-fixed at 4% formaldehyde for 4 hours. The tissues were dehydrated with graded ethanol at the concentration of 60 to 100%, cleared in xylene, filtrated in paraffin wax, and embedded in paraffin wax for 48 hours. Sections (6 μm thick) were obtained using a freezing sliding microtome. After deparaffinization, the sections were stained with Nissl solution (cresyl violet acetate) for 15 minutes, and then dehydrated and mounted on the slides. The morphology of hippocampal neurons in CA1 region was examined, and the number of hippocampal neurons was counted in a 1 mm length of the middle portion of hippocampal CA1 region under bright-filed microscopy.

After irradiation, cultured hippocampal neurons were treated with 1 μg/ml DAPI in methanol, and incubated at 37°C for 15 minutes. The staining solution was washed off with methanol and PBS. The morphology and number of hippocampal neurons were examined under fluorescent microscope. Roscovitine, Cdk5 inhibitor, was added into the cells 15 min before irradiation.

DAPI used in this study contains DNA-specific dye. This dye can pass through cell membranes, apoptosis increases cell membrane permeability and uptake of DAPI, leaving a blue stain. Apoptotic cells show irregular edges around the nucleus, chromosome concentration in the nucleus, heavier coloring, and, with nuclear pyknosis, an increased number of nuclear body fragments. For these reasons, the intensity of the fluorescence can help identify more accurate apoptotic cells when using DAPI images than TdT-Mediated dUTP Nick-End Labeling (TUNEL) staining, as TUNEL technique used in our previously unpublished studies has showed less accurate apoptotic cells.

### Statistical analysis

Analyses were performed using SPSS 13.0. All values were presented as mean and standard deviation. Student *t* test or analysis of variance (ANOVA) was used to compare the difference. Probability values less than 0.05 were considered statistically significant.

## Competing interests

The authors declare that they have no competing interests.

## Authors’ contributions

AMS and CGL carried out the studies, and drafted the manuscript. YQH carried out the immunoassays. QLL and QX participated in the design of the study and performed the statistical analysis. YWY conceived of the study, and participated in its design and coordination and helped to draft the manuscript. All authors read and approved the final manuscript.
